# Characterizing exons and introns by regularity of nucleotide strings

**DOI:** 10.1186/s13062-016-0108-7

**Published:** 2016-02-08

**Authors:** Tonya Woods, Thanawadee Preeprem, Kichun Lee, Woojin Chang, Brani Vidakovic

**Affiliations:** H. Milton Stewart School of Industrial & Systems Engineering, Georgia Institute of Technology, 765 Ferst Drive NW, Atlanta, 30332 USA; Faculty of Pharmaceutical Sciences, Ubon Ratchathani University, Ubon Ratchathani, Thailand; Hanyang University, Seoul, Korea; Seoul National University, Seoul, Korea

**Keywords:** Regularity, Cumulative evolutionary slope, Exons, Introns, Wavelets

## Abstract

**Background:**

Translation of nucleotides into a numeric form has been approached in many ways and has allowed researchers to investigate the properties of protein-coding sequences and noncoding sequences. Typically, more pronounced long-range correlations and increased regularity were found in intron-containing genes and in non-transcribed regulatory DNA sequences, compared to cDNA sequences or intron-less genes. The regularity is assessed by spectral tools defined on numerical translates. In most popular approaches of numerical translation the resulting spectra depend on the assignment of numerical values to nucleotides. Our contribution is to propose and illustrate a spectra which remains invariant to the translation rules used in traditional approaches.

**Results:**

We outline a methodology for representing sequences of DNA nucleotides as numeric matrices in order to analytically investigate important structural characteristics of DNA. This representation allows us to compute the 2-dimensional wavelet transformation and assess regularity characteristics of the sequence via the slope of the wavelet spectra. In addition to computing a global slope measure for a sequence, we can apply our methodology for overlapping sections of nucleotides to obtain an “evolutionary slope.” To illustrate our methodology, we analyzed 376 gene sequences from the first chromosome of the honeybee.

**Conclusion:**

For the genes analyzed, we find that introns are significantly more regular (lead to more negative spectral slopes) than exons, which agrees with the results from the literature where regularity is measured on “DNA walks”. However, unlike DNA walks where the nucleotides are assigned numerical values depending on nucleotide characteristics (purine-pyrimidine, weak-strong hydrogen bonds, keto-amino, etc.) or other spatial assignments, the proposed spectral tool is invariant to the assignment of nucleotides. Thus, ambiguity in numerical translation of nucleotides is eliminated.

**Reviewers:**

This article was reviewed by Dr. Vladimir Kuznetsov, Professor Marek Kimmel and Dr. Natsuhiro Ichinose (nominated by Professor Masanori Arita).

**Electronic supplementary material:**

The online version of this article (doi:10.1186/s13062-016-0108-7) contains supplementary material, which is available to authorized users.

## Background

### Structures of eukaryotic genomes

A genome is a complete set of genetic material for an organism. Except for RNA viruses, genomes are made of DNA (consisting of A, C, G, and T nucleotides). While the genomes of prokaryotes are gene-rich with a few noncoding regions, eukaryotic genomes contain longer intergenic sequences. In fact, only a small fraction (<2 *%*) of the eukaryotic genomes are comprised of genes, but the majority of those genes code for proteins [1]. In general, protein synthesis requires two steps: transcription and translation. In eukaryotes, a pre-mRNA is synthesized from a DNA template during the transcription process. Later, the pre-mRNAs undergo extensive modifications, including the splicing out of noncoding regions (introns) and the joining of coding regions (exons) to produce mature mRNAs. In the translation step, the mature mRNAs are translated into proteins.

As introns do not appear in the mature mRNA, they were originally thought to carry unimportant sequences [2]. However, introns are now known to have biological functions [3]. They harbor a variety of regulatory elements such as untranslated RNAs and splicing control elements, which regulate the mRNA processing and allow alternative splicing, a mechanism leading to greater variability of gene products (proteins). In addition to sequence motifs of introns that are driven by their roles during transcription, introns also carry features that associate with exon structure. Zhu and coworkers analyzed the variability of intron-exon architecture across many genomes and detected that some intron properties such as length, ordinal position, and *GC* content are correlated with the exon structure. One notable correlation was observed between the *GC* content of an intron and its flanking exons [4].

Local irregularities along a DNA strand, compared to surrounding regions, have been associated with biological functionality (i.e. coding for proteins and functional RNAs). Haimovich [5] suggested that if pattern irregularities are observed in introns, it may indicate biological significance of specific intron regions. In addition to presence/absence of biological functionality, variation of long-range correlation levels of different genomic regions has been used in the study of origin of genes and introns [6] and the effects of mutation accumulations or various evolutionary genomic events (replication slippage, recombination, translocation, and transposition) on sequence regularity [7].

### Previous work on translating DNA nucleotides to numerical sequences

Translating DNA into a numeric form has been approached in many ways and has allowed researchers to investigate the properties of protein-coding sequences and noncoding sequences. Peng et al. [8] first mapped nucleotide sequences onto a “DNA walk” in which the walker moves along the DNA sequence, stepping up (*u*(*i*)=+1) if a pyrimidine occurs and stepping down (*u*(*i*)=−1) if a purine occurs. They characterize the fractal landscape of DNA quantitatively using the mean fluctuation function *F*(*l*), defined by

(1) $$\begin{array}{@{}rcl@{}} F^{2}(l) = \overline{\left[\Delta y(l) - \overline{\Delta y(l)}\right]^{2}}, \end{array} $$

where *y*(*l*) for a given *l*, defined to be $y(l) = \sum _{i=1}^{l} u(i)$, is a trajectory of the DNA walk and *Δy*(*l*)=*y*(*l*_0_+*l*)−*y*(*l*_0_), where *l*_0_ is a given position in over all positions in the gene. In (1), the overline stands for the average. Segments of DNA which are uncorrelated or only short-range correlated have *F*(*l*)∼*l*^1/2^, while segments with long-range correlation have *F*(*l*)∼*l*^*α*^ (*α*≠1/2). For the sequences studied, Peng et al. found long-range correlations (regularity) in intron-containing genes and in non-transcribed regulatory DNA sequences, but not in cDNA sequences or intron-less genes. A similar large scale study has also shown the presence of long-range correlations for noncoding sequences [9]. More recently, a group of researchers attempted to quantify the degree of non-stationarity of DNA sequences through rescaled range analysis [10]. They used the rescaled range of a segment to estimate its Hurst exponent, a measure of self-similarity. Their methodology illustrated, in agreement with earlier results, that exons (coding regions) have lower Hurst exponents than introns (noncoding regions).

Numeric conversion methodologies other than the DNA walk have also been proposed. Stoffer et al. [11] approached the problem of scaling in nucleotide sequences by using so-called “spectral envelopes”. The idea behind this methodology is to find numerical scaling values to assign to each category of nucleotide which will maximize the variance of the resulting stationary time series’ spectral density across frequencies relative to the total variance. Another more simplistic approach is to represent DNA with four separate binary indicator sequences corresponding to the four nucleotide bases [12–14]. Often binary indicator sequences are grouped according to their chemical structures for statistical analysis [15–17].

One interesting numeric mapping solution uses the concept of symbolic autocorrelation [18]. Given the sequence of nucleotide symbols *x*_*i*_, its symbolic autocorrelation is the numeric sequence *r*_*k*_

$$\begin{array}{@{}rcl@{}} r_{k} = \sum\limits_{i=0}^{n-1} d\left(x_{i}, x_{i+k}\right), \end{array} $$

where for any two symbols *a* and *b*,

$$ d(a,b) =\left\{ \begin{array}{ll} 1, & \text{if}\,\, a=b\\ 0, & \text{if}\,\, a \neq b \end{array} \right. $$

Then the discrete Fourier transform of this autocorrelation is the spectrum of the symbolic data.

Our approach in this paper is to translate sequences of DNA into numeric matrices to be analyzed via wavelet analysis. We define the *cumulative evolutionary slope* of a sequence and show how it can be used to assess the scaling in nucleotide sequences. An advantage of the proposed method is its invariance with respect to assignment of nucleotides to their numerical values. Unlike the DNA walks where the nucleotides are assigned numerical values depending on nucleotide characteristics (purine-pyrimidine, weak-strong hydrogen bonds, keto-amino, etc.) or other spatial assignments, the proposed scaling measure is invariant to the assignment of nucleotides. Thus, ambiguity in numerical translation of nucleotides is eliminated.

## Methods

### From ACGT to numbers

#### About wavelet transforms and regularity measures

In this section we briefly and informally discuss wavelet transforms and terminology necessary for understanding the introduced methodology.

Wavelet transforms of signals/images are atomic representations in terms of orthogonal basis functions, similar to Fourier transforms. Orthogonal basis functions are formed by integer shifts and dilations of two fixed functions: a wavelet function and a scaling function. Thus, each basis function in the wavelet representation carries information about location and scale features of the signal/image. As such, the wavelet transforms are often dubbed time/scale decompositions.

Operationally, wavelet transforms are implemented by fast filtering according to Mallat’s algorithm. Each wavelet can be connected with a pair of filters: smoothing and differencing, and wavelet transforming of a signal/image amounts to repetitive filtering. The pair of wavelet filters are called quadrature mirror filters. More information on wavelet constructions, decompositions and connections with filtering can be found in an excellent monograph [19].

Since the wavelet transforms are linear and orthogonal, they also can be represented by sparse orthogonal matrices consisting of elements of quadrature mirror filters. Matrix representations of wavelet transforms are convenient for relatively short signals; the transform is conducted by multiplying the input with a wavelet matrix. More about forming wavelet matrices from the filter elements is described in [20].

As we indicated, wavelet transforms lead to coefficients (numerical values) representing the nature of a given signal at different locations/resolutions. These coefficients may be used to form the *wavelet-based spectra* of the signal, showing the relationship between the resolution of the signal and the averaged magnitudes of the coefficients. By assessing the wavelet-based spectra, we may better understand the mathematical characteristics of the overall signal. If the *energies* (an engineering term for squared coefficients in the wavelet decomposition) decay regularly, this signifies scaling in the data, meaning all resolutions contribute to the overall observed phenomenon. In this case, a measure of regularity can be calculated as the rate of energy decay. More precisely, if the logarithms of average energies in different scales decay linearly with the scale index, then the slope of this decay is describing the regularity of the original signal/object. Thus the spectral slope of the wavelet-based spectra can precisely measure the degree of a signal’s regularity.

For details about the wavelet-based spectra and its application to assessing regularity of signals/images we direct the reader to [19, 21].

#### Translating to matrices via assignment of unit vectors

Suppose that a nucleotide sequence of length *N* is encoded to the index matrix 4×*N* such that *A* is coded as *e*_1_=(1,0,0,0)^′^, *C* as *e*_2_=(0,1,0,0)^′^, *G* as *e*_3_=(0,0,1,0)^′^ and *T* as *e*_4_=(0,0,0,1)^′^. Denote this matrix with *Y*. For example,

$$\begin{array}{@{}rcl@{}} GATCTCT \ldots \longrightarrow Y = \left[ \begin{array}{ccccccccc} 0 & 1 & 0 & 0 & 0 & 0 & 0 & \\ 0 & 0 & 0 & 1 & 0 & 1 & 0 & \dots \\ 1 & 0 & 0 & 0 & 0 & 0 & 0 & \\ 0 & 0 & 1 & 0 & 1 & 0 & 1 & \\ \end{array} \right]. \end{array} $$

Assume also that *N* is a power of 2 for implementational purposes. Define *Y*^∗^ as the matrix formed by accumulating across the rows of *Y*. Continuing the above example,

$$\begin{array}{@{}rcl@{}} Y^{*} = \left[\begin{array}{ccccccccc} 0 & 1 & 1 & 1 & 1 & 1 & 1 & \\ 0 & 0 & 0 & 1 & 1 & 2 & 2 & \dots \\ 1 & 1 & 1 & 1 & 1 & 1 & 1 & \\ 0 & 0 & 1 & 1 & 2 & 2 & 3 & \\ \end{array} \right]. \end{array} $$

If *W*_4_ is a 4×4 matrix corresponding to Haar wavelet transform of depth 2, then

$$\begin{array}{@{}rcl@{}} W_{4} = \left[ \begin{array}{cccc} 1/2 & 1/2 & 1/2 & 1/2 \\ 1/2 & 1/2 &-1/2 & -1/2\\ \sqrt{2}/2 & -\sqrt{2}/2 & 0 & 0 \\ 0 & 0 & \sqrt{2}/2 & -\sqrt{2}/2 \\ \end{array} \right]. \end{array} $$

Define *D*=*W*_4_×*Y*^∗^ to be a matrix in which the columns of *Y*^∗^ are Haar transformed. In *D* the accumulated unit vectors are replaced by Haar orthogonal vectors that are columns of *W*_4_. This transforms the sparse *Y* to a more dense representation, *D*. For example,

$${\fontsize{8.8}{6} \begin{aligned} GATC \dots \longrightarrow Y^{*} \longrightarrow D = \left[ \begin{array}{ccccc} 1/2 & 1 & 3/2 & 2 & \dots \\ -1/2 & 0 & -1/2& 0 & \dots \\ 0 & \sqrt{2}/2 & \sqrt{2}/2 & 0 & \dots \\ \sqrt{2}/2 & \sqrt{2}/2 & \sqrt{2} & \sqrt{2} & \dots \\ \end{array} \right]. \end{aligned}} $$

Transform the rows of *D* using wavelet transform that has the depth ≥2 to obtain matrix *Z* of size 4×*N*. In matrix notation,

$$\begin{array}{@{}rcl@{}} Z = W_{4} \times Y^{*} \times W_{N}', \end{array} $$

where *W*_*N*_ is an *N* by *N* matrix. Now *Z* is the 2-D scale-mixing wavelet transform of *Y*^∗^, see Ramirez et al. [22].

The wavelet basis generating matrix *W*_*N*_ can be arbitrary, but the Haar is most natural since the rows of *D* are piecewise constant. Also, when *N* is large (say, >2^11^) the transformation by *W*_*N*_ is done by Mallat’s algorithm instead of direct matrix multiplication.

**Fig. 1:**
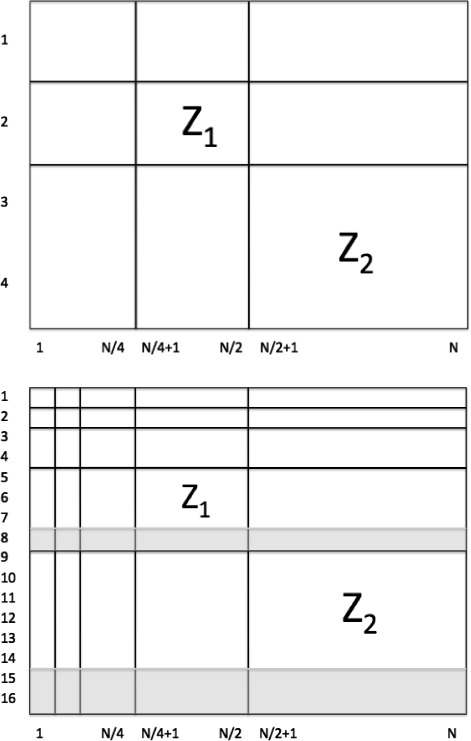
Log-spectral slope hierarchies. Illustration of submatrices *Z*_1_ and *Z*_2_, the levels of details forming the hierarchy for defining the log-spectral slope, in the original (top) and invariant (bottom) procedures

A submatrix of *Z*, *Z*_2_=*Z*(3 : 4,*N*/2+1:*N*) corresponds to the finest details of scale-mixing 2-D wavelet transform while *Z*_1_=*Z*(2,*N*/4 + 1 : *N*/2) is the next coarser detail level. Since one dimension of *Z* is 4, there are only 2 levels of details *Z*_1_ and *Z*_2_ that form the hierarchy for defining the log-spectral slope (see Fig. 1 (top)).

Unlike the traditional wavelet log-spectra that is based on log average energies at several levels, usually ≥4 depending on size of data, here we have only two spectral points – generated by *Z*_1_ and *Z*_2_, and the slope is estimated from that pair. Log-spectral slope, or simply *slopes* is defined as

$$\begin{array}{@{}rcl@{}} s = \log\left(\overline{Z_{2}^{*2}}\right) - \log\left(\overline{Z_{1}^{*2}}\right), \end{array} $$

where $\overline {A^{*2}}$ is the average entry of Hadamard square *A*∗*A* for arbitrary matrix *A*. (Since Hadamard square squares each entry of matrix, the $\overline {A^{*2}}$ is the mean of the squared entries of *A*.) The slope measures the change in energy between adjacent dyadic levels of the transformed matrix. If equal to 0, then the energies are comparable, and this case corresponds to independent random nucleotides. A negative slope indicates presence of regularity. In the 1-dimensional case (e.g. time series), high regularity means having long-term positive autocorrelation. In other words, a high value in the series will probably be followed by another high value, and the values a long time into the future will also tend to be high. This concept of regularity may similarly be extended to the 2-dimensional case. In contrast, a positive slope indicates an “explosion of energy” at finer levels of detail, indicating “zig-zagging” irregularities in the sequence. This simply means that the average magnitudes of wavelet coefficients are larger at small scales which translates to high anti-persistence in signal behavior.

#### Equivalence classes

Consider a particular DNA subsequence, say *ACGT*. Let the sequence be assigned to {*e*_1_,*e*_2_, *e*_3_,*e*_4_}, as defined in the previous section, in this order and the resulting slope be *s*. This sequence consists of two pairs of dinucleotides, *AC* and *GT*. The slope is not changed if the nucleotides within each pair are permuted and if the pairs themselves are permuted. For example, the same slope is obtained by assigning *ACTG, CAGT, CATG, GTAC, GTCA, TGAC*, and *TGCA* to {*e*_1_,*e*_2_,*e*_3_,*e*_4_}.

Furthermore, the 24 permutations of *ACGT* lead to

$$\begin{array}{@{}rcl@{}} \frac{4!}{2! ~ 2! ~ 2!} = 24/8 = 3 \end{array} $$

equivalence classes that result in three different slopes. Table 1 lists the assignments in these equivalence classes.

**Table 1:** Three equivalence classes of assignments of nucleotides to unit vectors that lead to three different slopes, *s*_1_, *s*_2_ and *s*_3_

*s*_1_	*s*_2_	*s*_3_
*ACGT*	*AGCT*	*ATCG*
*ACTG*	*AGTC*	*ATGC*
*CAGT*	*GACT*	*TACG*
*CATG*	*GATC*	*TAGC*
*GTAC*	*CTAG*	*CGAT*
*GTCA*	*CTGA*	*CGTA*
*TGAC*	*TCAG*	*GCAT*
*TGCA*	*TCGA*	*GCTA*

We notice that each class represents a nucleotide characteristic. The class of *AC* and *GT* corresponds to amino and keto, that of *AG* and *CT* purines and pyrimidines, and that of *AT* and *CG* weak hydrogen bonds and strong ones.

#### Translation invariant procedure

In order to be an effective measure of regularity, the slope should not depend on the way in which nucleotides are assigned to unit vectors. The simplest way to find a representative slope is to average the slopes *s*_1_, *s*_2_ and *s*_3_, where the subscript denotes the equivalence class. An alternative (and better) way is to assign nucleotides to unit vectors based on representatives from each of the equivalence classes and stack these unit vectors together. For example, *ACGT, AGCT,* and *ATCG* could each be assigned to (*e*_1_,*e*_2_,*e*_3_,*e*_4_), or equivalently, any other representative triple from the three columns in Table 1. Ultimately, each nucleotide is assigned a vector of length 12. For example, if *ACGT, AGCT,* and *ATCG* are used, *C* would correspond to (0 1 0 0 0 0 1 0 0 0 1 0)^′^.

Following this procedure, *Y* becomes a 12×*N* matrix. In implementations, a matrix *Y* with 16 rows is used where the last four rows are arbitrary (say zeros) and serve only to fulfill the power of 2 requirement for operational use of wavelets. Since the Haar basis is used, this padding does not leak to the relevant coordinates. Then *Y*^∗^ is computed by accumulating across the columns of *Y*. If *Z* is produced using the Haar wavelet, and the slope is estimated, this slope is invariant with respect to permutation of coding. In matrix notation,

$$\begin{array}{@{}rcl@{}} Z = W_{16} \times Y^{*} \times W_{N}'. \end{array} $$

In this case, the submatrices of *Z* defining the slope are *Z*_2_=*Z*(9:14,*N*/2+1:*N*) and *Z*_1_=*Z*(5:7,*N*/4+1:*N*/2) (see Fig. 1 (bottom)). This resulting slope is invariant with respect to the assignment of unit vectors *e*_1_ through *e*_4_ to the nucleotides, as long as the assignments from each of the three equivalence classes are used to form matrix *Y*.

#### Cumulative evolutionary slope

Take a submatrix $Y_{k}^{*}$ of size 16×*k* and shift it along the nucleotide. Transform it to *Z*_*k*_ as

$$\begin{array}{@{}rcl@{}} Z_{k} = W_{16} \times Y_{k}^{*} \times W_{k}', \end{array} $$

and find corresponding slopes. This series of slopes is the *cumulative evolutionary slope* for the sequence. We emphasize that for fixed submatrix this gives a single slope. The slope dynamically changes as the submatrix slides along the nucleotide sequence. The term evolutionary has no biological content, it indicates that the slope changes in the process.

**Fig. 2:**
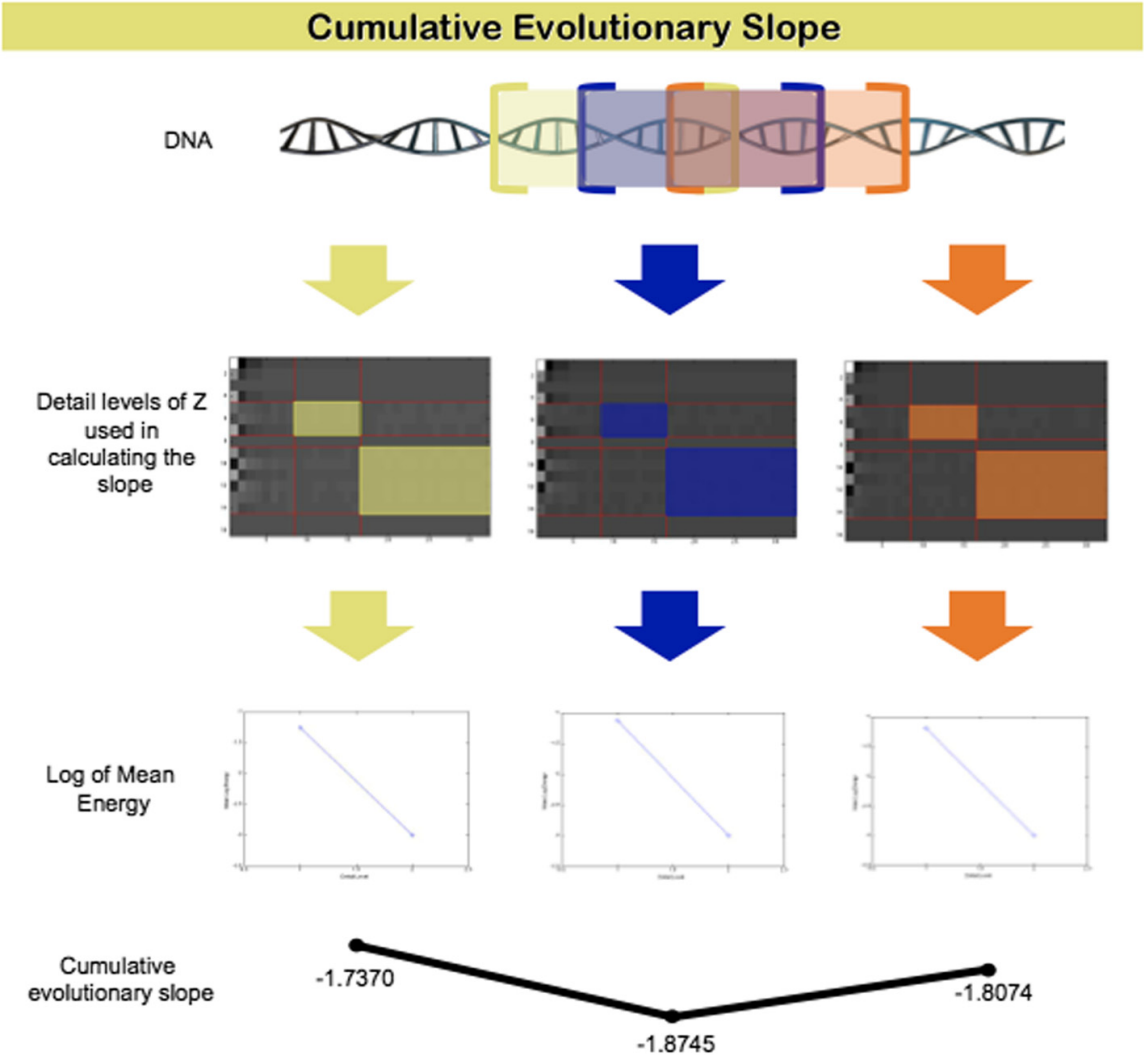
Cumulative evolutionary slope calculation. Overlapping sequences of DNA nucleotides are represented as matrices, the scale-mixing wavelet transformation is applied to these matrices, log average energies are computed for the shown detail levels, and slopes are calculated

If the Haar wavelet basis is used, the shifts should be with steps divisible by 4. Values of *k* that are too small lead to noisy evolutionary slope, while values too large lead to loss of locality. As an illustration of the calculation of the cumulative evolutionary slope, Fig. 2 shows three shifts in a nucleotide sequence generate matrix *Z*_*k*_ and associated slopes.

### Application

To illustrate our methodology, we study regularity characteristics of exons and introns from the honeybee’s first chromosome. The average *GC* contents for the sequences analyzed are 32.16 % for exons and 24.59 % for introns, and the average exon and intron lengths are 239 nucleotides and 1,791 nucleotides respectively. A comprehensive study of the honeybee genome indicates honeybee genes are much depleted in *C* and *G* nucleotides (gene-averaged *GC* content of 29 %) [23].

#### Data

The reference DNA sequence from chromosome 1 (linkage group LG1) of the honeybee representative strain, *Apis mellifera* Amel_4.5, is used in our analysis. We obtained the sequence from the NCBI Genome Database (http://www.ncbi.nlm.nih.gov/genome, retrieved November 7, 2013). The DNA sequence of chromosome 1 (NC_007070.3) contains a total of 29,893,408 base pairs. The overall *GC* content is 31.20 %.

For each gene, we use the NCBI annotations of coding sequences (CDSs) to identify exon-intron boundary. Then, we parse the gene sequences into coding and noncoding regions. There are 1,669 genes in the honeybee first chromosome, but only 376 genes contain known locations of CDSs. Therefore, we limit our analysis to this subset of genes. DNA has a double helix structure with a complementary nucleotide sequence on each strand. To represent the duplex structure as a single strand, we treat all gene sequences on the reverse strand as if they were located on the forward strand. Specifically, we correct the gene direction and represent the gene sequences with their complementary sequences. This way, every gene can be read from left to right during the DNA analysis. All sequence processing tasks were performed with in-house Perl scripts, and subsequent analysis was performed using MATLAB [see Additional files 1 and 2].

## Results and discussion

### Comparing regularity of honeybee and simulated DNA

To assess regularity characteristics of the honeybee DNA, we first compute global slope measures. Take as an example a single gene sequence from the honeybee’s first chromosome:



As the length of this sequence is 877 nucleotides but computing global slope requires the length to be a power of 2, we truncate the sequence to include the first 512 nucleotides and calculate the global slope of this shortened sequence. We also generate 10,000 random DNA-like sequences, generated from the multinomial distribution where the proportions of nucleotides match those of the original gene sequence (in this case: 33.03 % *A*, 16.28 % *C*, 19.84 % *G*, 30.85 % *T*). These simulated DNA-like sequences serve as a control for examining overall regularity characteristics of the actual DNA sequence.

**Fig. 3:**
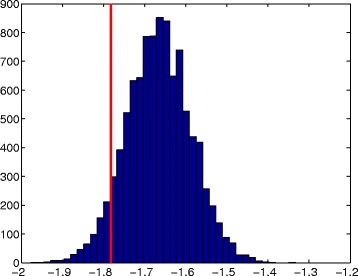
Comparing regularity of actual and simulated DNA sequences - global. Global slope for the honeybee DNA (red line at −1.7825) sequence and empirical distribution of slopes for 10,000 simulated random DNA-like sequences of length 2^9^ (ASL =0.0676)

Figure 3 plots the bootstrap distribution of global slopes from simulated DNA-like sequences as a histogram and plots the global slope from the honeybee DNA sequence as a vertical red line. Comparing the actual and simulated DNA sequences, the actual DNA sequence’s slope falls in the left tail of the control distribution. The achieved significance level (ASL) for this simulation (area in the bootstrap distribution left of the red bar) is 0.0676. This result indicates that the honeybee nucleotide sequence is generally more regular than the randomly generated DNA based on the bootstrap distribution of the slopes from DNA-like sequences. We also conduct a permutation test by taking the DNA sequence and permuting it 20,000 times. For each permutation we find the spectral slope. The achieved significance rate (empirical *p*-value) for this test is 0.0638 [see Additional file 3].

**Fig. 4:**
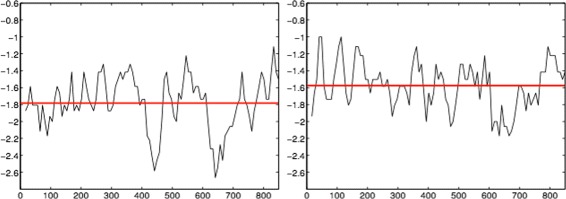
Comparing regularity of actual and simulated DNA sequences - cumulative evolutionary. Honeybee cumulative evolutionary slope for gene “*LOC100577807*” (*left*); Cumulative evolutionary slope for a random DNA-like sequence (*right*); In both cases window size was 2^5^

Figure 4 compares the cumulative evolutionary slope of the honeybee DNA to that of a simulated random DNA-like sequence. Note that this time, the gene sequence only has to be truncated so that the length is divisible by the step size. In each plot, the red horizontal line indicates the overall mean of the slopes. The average cumulative evolutionary slope for the honeybee gene is around −1.8, while the average cumulative evolutionary slope for the simulated sequence is around −1.6. These plots support the claim that the actual honeybee DNA is more regular than the randomly generated DNA.

**Fig. 5:**
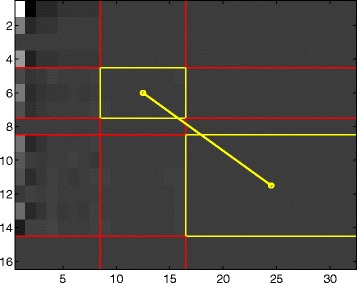
Detail levels in slope calculation. An illustration of detail levels of Z used in the slope calculation for window size 32, honeybee DNA

Figure 5 shows *Z*, the 2-D scale mixing wavelet transform of *Y*^∗^, with highlighted detail levels used in the local slope calculation. As previously stated, rows 8, 15, and 16 are excluded in the slope calculation due to the arbitrary rows of zeros added to the original numeric matrix in order to satisfy the power of 2 requirement for operational use of wavelets.

### Comparing regularity of exons and introns

What happens when we compare sequences of exons and introns within the honeybee? Considering 376 genes for which the coding designations are known, we plot the cumulative evolutionary slope for each gene sequence.

We label sections of the plot as introns, exons, or a combination of the two. We find it necessary to define combination regions due to our evolving slope methodology. Since we calculate local slopes for overlapping regions of DNA nucleotides of fixed length, some regions of nucleotides contain both exons and introns. Without defining combination regions, the local slopes of exons and introns are averaged together, causing ambiguity in the results.

**Fig. 6:**
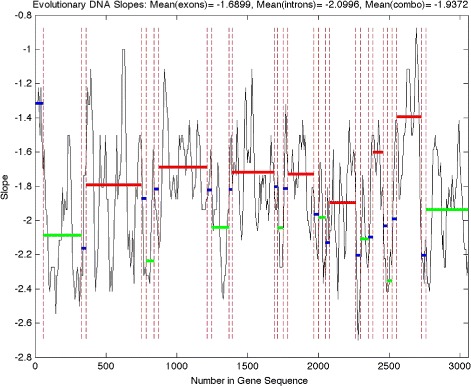
Honeybee cumulative evolutionary slope for gene “*LOC408625*” on the first chromosome. *Solid red line*: Average slope for a coding sequence (exons), *solid green line*: Average slope for a noncoding sequence (introns), *solid blue line*: Average slope for a sequence with a mixture of coding and noncoding, *dotted red line*: Division between type (exons, introns, combination) of region

Figure 6 shows the cumulative evolutionary slope for one of the genes on the first chromosome. The dotted red lines show divisions between types of regions (exons, introns, combination). Solid red lines indicate average slope values for exons, solid green lines indicate average slope values for introns, and solid blue lines indicate average slope values for sequences with a mix of exons and introns. We choose a window size of 2^5^ with step size 2^3^ to capture characteristics of coding sequences, which are sometimes only around 50 nucleotides in length.

**Fig. 7:**
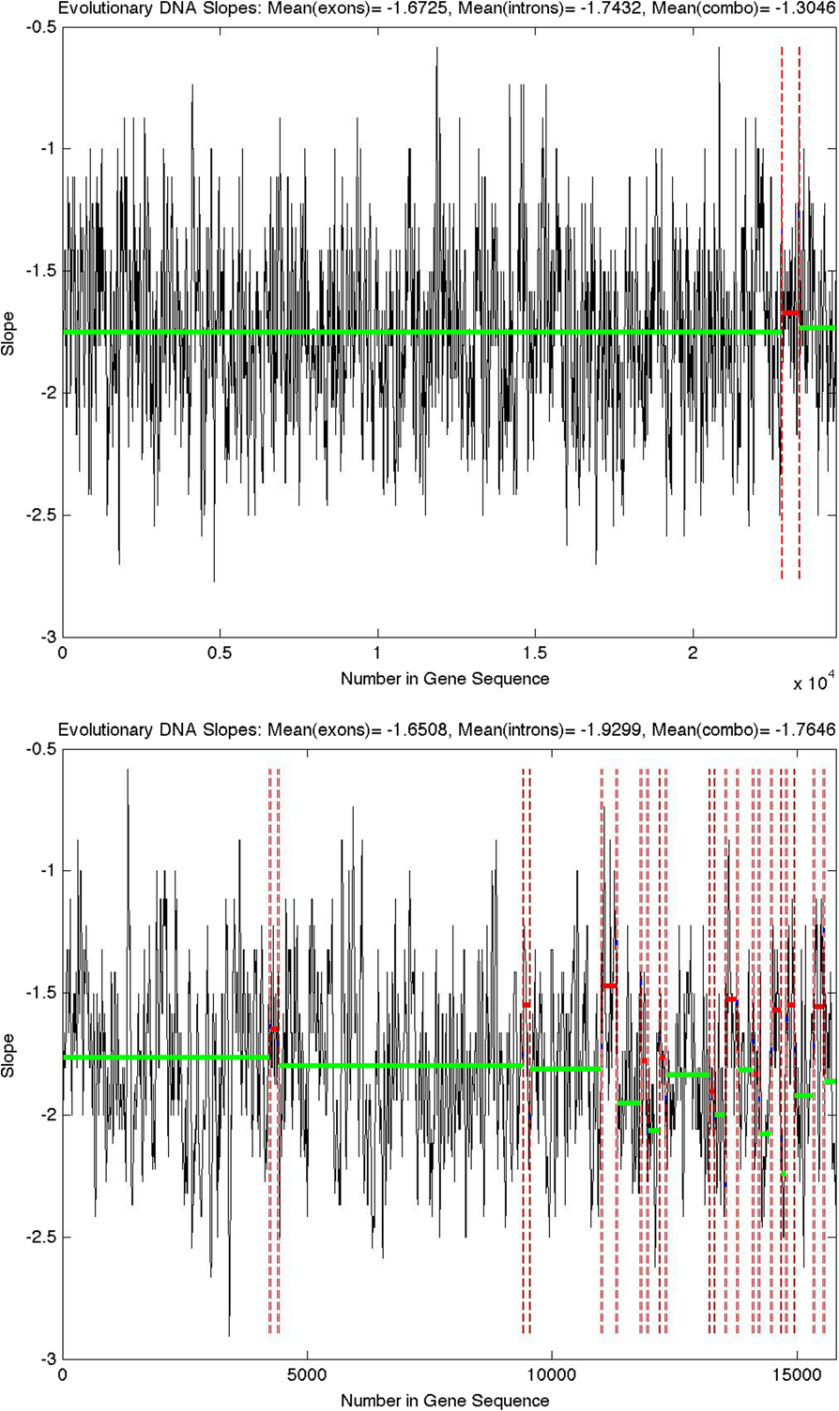
Honeybee cumulative evolutionary slope for genes “*ARD1*” *(top)* and “*DAT*” *(bottom)* on the first chromosome. *Solid red line*: Average slope for a coding sequence (exons), *solid green line*: Average slope for a noncoding sequence (introns), *solid blue line*: Average slope for a sequence with a mixture of coding and noncoding, *dotted red line*: Division between type (exons, introns, combination) of region

It is evident from Figs. 6 and 7 that exons are associated with less negative slopes (more irregular) while introns are associated with more negative slopes (more regular). Figure 6 gives an example of results from a shorter gene on the first chromosome, while Fig. 7 gives two examples of results from longer genes with long introns (characteristic of the honeybee genome, as discussed previously) on the first chromosome. Similar results are obtained for the other genes on the first chromosome. The average cumulative evolutionary slope across all 376 genes for exons and introns are −1.7395 and −1.8410, respectively (sample sizes: 1974, 2296). A two sample t-test reveals this difference in slopes to be highly significant (t-statistic =17.4, *p*-value ≈0). Note that some coding, noncoding, or combination regions were excluded due to infinite slopes. These infinite slopes are due to zero-valued mean energies for the finest detail level (see Fig. 5). In our analysis, 69 genes have at least one section with infinite slope. Interestingly, of those 69 genes containing at least one infinite slope, 57 have infinite slopes for intron sections only.

**Fig. 8:**
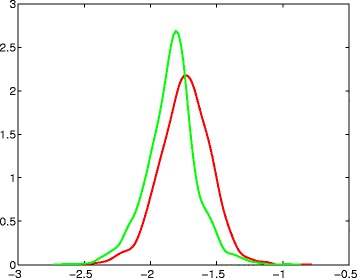
Kernel density estimates. Kernel density estimates for cumulative evolutionary slopes, shown on the horizontal axis, of exons (*red*) and introns (*green*)

To better understand the level of separation between the slopes of exons and introns, we find kernel density estimates computed at 100 points covering the range of the data (Fig. 8). In addition, we compute the slope value (*s*^∗^=−1.735) to discriminate between exons and introns which maximizes the Youden Index, defined as

$$\begin{array}{@{}rcl@{}} J=sens+spec-1, \end{array} $$

where *sens* is the sensitivity and *spec* is the specificity of the classification.

Table 2 summarizes the classification results for *s*^∗^=−1.735. The sensitivity and specificity of this classification are 50 % and 76.2 %, respectively, and the overall accuracy is 64.1 %. The Matthews correlation coefficient (MCC) is 0.27. Our intention in presenting these classification results is not to suggest that this procedure is meant for classifying sequences of nucleotides as either exons or introns. Rather, our intention is to present the level of separation between the cumulative evolutionary slopes of exons and introns, as a mechanism for characterizing their regularity properties.

**Table 2:** Classification results for the cut-off slope value, *s* ^∗^=−1.735, which maximizes the Youden Index

	Exons	Introns	Total
Slope >*s* ^∗^	987	546	1533
Slope ≤*s* ^∗^	987	1750	2737
Total	1974	2296	4270

Our analysis of 376 genes from the first chromosome of the honeybee illustrates that introns are significantly more regular (lead to more negative spectral slopes) than exons. The biological explanation for this observed long-range correlation of introns (intron regularity) is not yet understood. The only known pattern that occurs repeatedly in every intron is the presence of splice donor and acceptor sites near the exon-intron boundary, and the inclusion of some regulatory elements towards the end of an intron. Our methodology uses a window size of 32 nucleotides for the cumulative slope calculation. Therefore, it is unlikely that the presence of splicing and regulatory motifs within the introns contributed to the reported regularity since they are most likely located in different windows.

As a side note, based on the works [6, 24], the presence of long-range correlations within the honeybee introns suggests their modern introns may be almost identical to the primordial ones and/or introns were subjected to large evolutionary constraints, thus maintaining their primitive periodicity. This hypothesis is supported by well studied organizational and evolutionary characteristics of the honeybee genome. The honeybee and two other insects, the fruitfly and the malaria mosquito, share about one thousand ancient genes (The Honeybee Genome Sequencing Consortium, 2006). This gene set was used to identify honeybee’s evolutionary rate from its ancestors. The study shows that honeybee retains the greatest fraction of ancient genes (∼ 33 *%*) and ancient introns (∼ 80 *%*). It highlights that honeybee’s genes appear to be ancient and that honeybee evolves more slowly than the fly and the mosquito [23]. Therefore our observations that the honeybee’s genes have low *GC* content and remarkably long introns with high regularity are in concordance with the high proportion of ancient genes and ancient introns.

## Conclusions

We have proposed a new method for representing sequences of DNA nucleotides as numeric matrices in order to analytically investigate regularity characteristics of DNA. Previous methods, where the nucleotides are assigned numerical values depending on nucleotide characteristics (purine-pyrimidine, weak-strong hydrogen bonds, keto-amino, etc.), lead to different regularity measures when different assignments are used. Our proposed method, however, results in a consistent regularity measure through the use of equivalence classes in forming the assignment procedure. Thus, subjectivity in numerical translation of nucleotides is eliminated.

We have also defined the *cumulative evolutionary slope* as a sequence of log-spectral slopes computed from submatrices of wavelet transformed matrices corresponding to overlapping sequences of DNA nucleotides. Shorter overlapping sequences result in noisier cumulative evolutionary slope, while longer overlapping sequences result in smoother cumulative evolutionary slope.

In order to illustrate our methodology, we have analyzed 376 genes from the first chromosome of the honeybee. We have found that introns are significantly more regular (lead to more negative slopes) than exons, which agrees with the results from the literature where regularity is measured on “DNA walks” [8–10]. Due to its objectivity in numerical translation, we suggest that this methodology may be extended to study the regularity characteristics of various DNA sequences in order to elaborate interesting patterns.

## Reviewer reports

### Response to referee team 1: Dr. Vladislav Bondarenko and Dr. Vladimir Kuznetsov

Major comment: *The manuscript (at least in some sections) is written in non-professional biological language with numerous examples of incorrect usage of terminology. Basic assumptions are often skipped or confused and not defined properly. The paper must be written with respect to the standards and stile acceptable for publication in the scientific journals. The actual novelty of this work is not clearly represented. Overall advantage of implementation of the method for a discrimination of intron and CDS sequences raises methodological and biological questions.*

The reviewer’s point is well taken. The intended audience of this paper are data scientists in the field of biology, and to this end we formalized our biological content. The “Background” chapter is completely rewritten and a paragraph added to “Results and discussion”

The novelty of this work is

1. The discovery of three equivalence classes, each class represents a nucleotide characteristic.
2. The use of cumulative evolutionary slope of equivalence classes allows the analysis of sequence regularity to be independent of nucleotide symbols or properties.
3. Cumulative evolutionary slopes were identified along a gene sequence using a sliding window approach. Average slopes for introns, exons, and the combined regions were calculated to represent the local regularity along a gene. Without defining the combination regions (regions spanning intron and exon boundaries), the slopes were getting averaged for portions of exons and introns together and the results were not as clear.
4. Many researchers regard the term “sequence regularity” differently. Some examined the nucleotide sequence landscape in term of “DNA walk” (Peng, 1992; Buldyrev et al., 1995). Some looked at the regularity across multiple exons and introns as the common distance among them (Ieviņa et al., 2006). In this work, we developed a cumulative evolutionary slope of equivalence classes to quantify sequence regularity within genes. In addition, we looked for regularity within an exon or intron itself.
5. The biological explanation for the observed long-range correlation of introns (intron regularity) is not yet understood. The only known pattern that occurs repeatedly in every intron is the presence of splice donor and acceptor sites near the exon-intron boundary, and the inclusion of some regulatory elements towards the end of an intron. Our methodology used the window size of 32 nucleotides for cumulative slope calculation. Therefore, it is unlikely that the presence of splicing and regulatory motifs within the introns will contribute to the reported regularity since they are most likely located in different windows.

*Comments and Questions*

**1.***What is an “overlapping section of nucleotides”? Please provide a definition for an “evolutionary slope” and how is it different from the slope of a wavelet spectra. How these terms are related to biological evolution and comparative genomics?*

The term “evolutionary slope” is common mathematical jargon for the slope that is calculated from subsequent sequences of along the genome. It has no connotation to biological evolution, the “evolutionary” relates to the moving of a subsequence of fixed length from which the slope is calculated. To avoid misunderstanding we added the following explanatory sentence:

The definition of evolutionary slope now reads as follows

Take a submatrix $Y_{k}^{*}$ of size 16×*k* and shift it along the nucleotide. Transform it to *Z*_*k*_ as

$$\begin{array}{@{}rcl@{}} Z_{k} = W_{16} \times Y_{k}^{*} \times W_{k}', \end{array} $$

and find corresponding slopes. This series of slopes is the *cumulative evolutionary slope* for the sequence. We emphasize that for a fixed submatrix this gives a single slope. The slope dynamically changes as the submatrix slides along the nucleotide sequence. The term evolutionary has no biological content, it indicates that the slope changes in the process.

**2.***The abstract should be more understandable. I did not understand a conclusion of the abstract: “Thus, subjectivity in numerical translation of nucleotides is eliminated”.*

The term subjectivity was changed to ambiguity. In traditional ways of assigning numbers to nucleotides, especially in DNA walks, 1 can be assigned to A,T, and -1 to C,G, or 1 to A,G and -1 to C,T, etc., leading to different slopes. Our proposed methods always leads to unique slope given the existence of three equivalence classes. We slightly modified the abstract.

**3.***In the first sentence of a “.*Background*” section, definition of a DNA molecule is incomplete and wrong. A phrase “In all eukaryotic species” is improperly used indicating that a DNA with a double helix structure consisting of purines and pyrimidines is characteristic to eukaryotes only, which is definitely a wrong statement.*

Thank you for the comment. We addressed the statement in the revised Background.

**4.***The same is related to a phrase “Protein synthesis of eukaryotes requires two steps: transcription and translation …”.*

Thank you for the comment. This we also addressed in the revised Background.

**5.***After that phrase authors should indicate that further they describe specifically mRNA processing. Post-transcriptional processing of different types of RNAs is also different.*

The section focuses on fundamental steps of central dogma in biology and is not intended to discuss the process in great details. The section had been revised accordingly.

**6.***“spliced mRNA” is not “a mature mRNA” yet, and these definitions should not be confused. Generally, authors should provide references to a competent scientific literature and other authors contribution to accompany every questionable statement.*

Thank you for the comment. The Background section has been revised to indicate that intron splicing is part of the mRNA processing step.

**7.***Is there the parameters of your method/program sensitive to a sequence source, its complexity length, and sequence errors in a dataset?*

Our method does not address this issue, but we selected one of the best complete genomic sequences at the time to ensure sequence errors are minimal. Also, our models are not *generative*, but rather *descriptive*.

**8.***Please specify where is and where is not a novelty of your method.*

We outline the novelty of our research in response to *Major Comment* above.

**9.***Please, provide a definition of a “real DNA’. This term seems not scientifically sound. Is an observed difference between cumulative evolutionary slope and ones of random sequences statistically significant?*

We agree with the reviewer, and term “real DNA” was dropped. The intended meaning was in the context of simulation of a DNA-like sequence based only on the observed nucleotide frequencies, as multinomial. Then we contrasted simulated and genuine DNA arrays, to which we referred as “real”.

**10.***Constructing the predictive model, the authors introduced a class of equivalence for the paired nucleotides. In fact, they proposed a code allowing the permutations within neighbouring nucleotides and between neighbouring sequence pairs. They said that “The slope is not changed if the nucleotides within each pair are permuted and if the pairs themselves are permuted”. Is there a biological basis for these assumptions?*

This is very interesting question. As the separation to equivalence classes and the slope invariance given the code representation from the class, this invariance is purely mathematical property.

**11.***Is your slope measure invariant to different kind of sequence truncation (at the beginning of a CDS, its end) or repeated loci)?*

The invariance relates only to the proposed coding of nucleotides. Sequence truncation, repeated loci are likely to induce change in slope.

**12.***How different length of truncated regions was normalized on a Figs.*6*and*7*? Is it scalable; if yes, please explain.*

Thanks for this question. The results shown at Figs. 6 and 7 did not require normalization and are scalable. Multiplying the wavelet coefficients with any scalar would affect the intercept of the linear fit of “energy” decay, but not the slope.

**13.***Please indicate your motivation and selection criteria for the organism of interest (honeybee). Why genes located on a chromosome 1 only have been used for analysis. Why Honeybee and why first chromosome? Results for other species might be important for evaluation of the method.*

Since our newly developed mathematical approach aims at quantifying sequence regularity within genes, we deemed it is necessary to emphasize the application to gene sequences of one organism at a time to see if the method is effective. During our preliminary analysis (November 2013), NCBI Genome Database provided a collection of reference sequences of many prokaryotic and eukaryotic organisms. We set to study the genome of eukaryotes as their genes comprise of exons and introns. Accurate exon-intron boundaries are crucial information in our work. Therefore, we must exclude any unfinished works or ambiguous sequences. Several filters were applied to the entire NCBI Genome Database. First, the sequence list was filtered such that only organisms with the status “complete” for their genome records were retained. This yields a list of reference genomes for 204 eukaryotic organisms. Next, we omitted any organisms that have multiple “representative strains” as gene annotations could be unclear. Then, we collected only genomic sequences with the accession prefix “NC” to ensure the sequence is completed, curated, and regarded as the reference assembly. The genome of Honeybee (*Apis mellifera*) was selected for several reasons:

- Honeybee is one of several model organisms. We expected that it is subjected to extensive studies, including gene annotations, and the evolution and characterization of the gene structure.
- Honeybee has one representative strain called “*Apis mellifera* Amel_4.5”.
- The reference sequences accession number for honeybee genome starts with the prefix “NC” which denotes complete genomic molecule, usually reference assembly. This provides a higher confident over partial genomes of many higher eukaryotes. em Due to time constraint, we did not analyze all genes in the honeybee genome. Instead, we focused on high confident annotated genes located on honeybee chromosome 1. This chromosome has the largest size and contains the highest number of genes.

**14.***What is an exact p-value for observed global slopes on Fig.*3*? Is it significant?*

Given the large sample sizes in calculating the spectra, the *p* values will be highly significant, given the overpowering. Moreover, the significance of *p*-values may not translate to an efficient classification procedure. Instead we opted classification measures (confusion table) to describe differences between coding and non-coding regions.

**15.***The estimations of specificity, sensitivity, robustness and reproducibility of the method must be reported.*

It was partially reported. On page 10 there is a statement: “Table 2 summarizes the classification results for *s*^∗^=−1.735. The sensitivity and specificity of this classification are 50 % and 76.2 %, respectively, and the overall accuracy is 64.1 %. The Matthews correlation coefficient (MCC) is 0.27.” Our analysis did not involve multiple chromosomes to assess the robustness and reproducibility.

**16.***It is important to demonstrate the advantages and disadvantages of your method in a comparison with alternative methods, for instance, the probabilistic models taking in to account triplet code and a relatively high evolutionary conservation of SDS sequences.*

We stated that alternative methods all agree in the fact that coding regions translate to more irregular numerical objects while the non-coding regions exhibit increased regularity. We are not aware of any methodology that will be comparable to the proposed on equal terms (multiple codes from equivalence classes, wavelet spectra from matrices/images).

**17.***Which programming language has been used?*

Our spectral analyses was done in MATLAB. All sequence processing tasks were performed with in-house Perl scripts.

**18.***The programming code and the instruction must be publicly available to research community according to open-source management of Biol. Direct journal.*

We plan to post MATLAB code and illustrative data form the 1st chromosome to illustrate the methodology. The suite will be posted on Jacket Wavelets web repository: http://gtwavelet.bme.gatech.edu/.

### Response to referee 2: Professor Marek Kimel

Thank you for your insightful comments.

**Page 4.***More information is needed for the reader to understand Haar transforms. As it is now, the background is missing which will distract many readers. An example of this is mentioning the Mallat’s algorithm with no discussion. On the same page the notion of the ’energy’ of spectral components is mentioned, which apparently is basic for understanding the principle on which the method is based. Also, define the Hadamard square.*

We gave a brief and informal introduction to wavelets. Mindful of the audience, we avoided technicalities. However, we directed interested readers to important references where detailed descriptions can be found.

Hadamard square is defined.

**Page 5.***Meaning of positive slope is defined as “explosion of energy”, indicating “zig-zagging” in the sequence. This is not informative. Similarly, it is unclear (to me at least) why the slope is invariant with respect to permutations within and among AC and GT and not with respect to other permutations?*

It would be very difficult and possibly not illuminating to formalize these statements. Since *positive slope* means that the average energy at the higher resolution level exceeds the energy in the coarser resolution level, positive slope signifies high irregularity. The signals with excess of irregularity (Hurst exponents between 0 and 0.5) are anti-persistent, informally described as zig-zagging. The following sentence is added: “... This simply means that the average magnitudes of wavelet coefficients are larger at small scales which translates to high anti-persistence in signal behavior.”

As regards the permutations of AC and GT within the pairs and among the pair, this is true only for *s*_1_ column of Table 1. Pairs AG and CT (column 2) and AT and CG (column 3) have the same properties. Note that one element of each column is needed for the invariant coding.

**Page 5.***The slope “should not depend” on the way..., or it simply “does not depend” (?) Also, the way vector are “stacked” so they form 12 x N Y-matrix, is not clear. Maybe a larger example would help. This seems fundamental for the method.*

Thanks for your comments. When we referred to slope that “should not depend” on the assignment of nucleotides to their numerical translations, we referred to generation of DNA walks, where spectra depends on how the nucleotides are coded. For example, the DNA walk A,T →+1, C,G →−1 has different spectra (and consequently spectral slopes) from the walk generated by A,G →+1, C,T →−1, even though the sequence is common. So our point was that the proposed method leads to no ambiguities and translation is unique.

**Page 7.***The two mechanisms of intron length increase are mentioned, but the description is cursory. Explain more or drop. The simulation-based test seems simplistic. A permutation test (one of many discussed in the literature) I think would be more on target.*

We are grateful for your suggestion and agree that permutation test here would be more on target. The way we did simulation is a variant of parametric bootstrap, where we selected samples from a multinomial distribution with prescribed probabilities of classes. As such, the proportions of nucleotides are matched only via expectations and not exactly. Permutation test will keep these proportions fixed.

We conducted the permutation test by taking the DNA string, permuting the string 20,000 times and for each permutation we found the spectral slope. The achieved significance rate for this test is 0.0638. Please see the included figure illustrating the results of the permutation test. Note that slope -1.7825 (red) falls in the left tail of the distribution. Therefore, given the fixed content of nucleotides, the autocorrelation among nucleotides, that is, spectral slopes in the wavelet domain, distinguish genuine genome from random sequences.

The following was added: “... We also conduct a permutation test by taking the DNA sequence and permuting it 20,000 times. For each permutation we find the spectral slope. The achieved significance rate (empirical *p*-value) for this test is 0.0638”.

**Page 8.***Please explain the biological interpretation of combination slopes. Also, referring to the study of Youden Index, please describe in more detail the sample that was used as a gold standard and reasons it is believed to be a gold standard. As a general remark, why is the honeybee genome considered? The most natural choice is probably human genome, and even better, several genomes of distantly related species.*

The scaling separation of exons and introns is a phenomenological observation, and we did not find an explanatory biological interpretation so far. Informally, one biologist from Georgia Tech postulates that increased regularity in exons may serve as a “protection of the code” because regular patterns are more easily repairable as compared to the irregular.

As regards the gold standard, the genome of honeybee is fully sequenced and we know exact locations of exons, introns, or combinations. We agree with you that human genome would be more interesting for the readership or some comparative analysis of genomes in distantly related species. We thank you for the suggestion and we hope to address this in the future.

### Response to referee 3: Dr. Natsuhiro Ichinose (nominated by Professor Masanori Arita)

*The matrix W*_4_ (or *W*_16_*) is not necessary to be the wavelet transformation. It would be important that W*_4_*provides some types of the “DNA walk”. For example, the second row of D corresponds to the DNA walk in which A or C implies +1/2 and G or T implies -1/2. Another row corresponds to another type of the DNA walk. Especially, the first row of D is redundant because D*_1*j*_=*j/2 for any sequences. Since the first row is not used in calculation of the scaling measure, it should be removed. Therefore, the authors should reconsider the matrix W*_4_ and *W*_1_*6. If there is an implementation reason such that W*_4_ or *W*_1_*6 is necessary to be the wavelet transformation, the authors should discuss that.*

The reviewers are correct. The matrices *W*_4_ (or *W*_16_) may not necessarily be the wavelet transform matrices. However, there are compelling reasons why these should be selected as wavelet matrices:

(i) Wavelet matrices are orthogonal and as such, they preserve the energy. This preservation of energy is critical for coherent definition of spectra,

(ii) The levels defined by wavelet matrices are dyadic and thus the energies decay among the levels in a calibrated manner. This means that if perfect mathematical monofractal is decomposed, the spectral slope will be −(2*H*+*d*) where *H* is Hurst exponent and *d* is dimension of the object. With non-wavelet matrices the counterpart of slope may not be connected with *H* in an obvious manner.

The first row in D, as the reviewer points out is redundant, but this row is not taken in the calculation of spectral slopes for it corresponds to scaling wavelet coefficients and not the detail. Only detail coefficients are used for spectral assessment.

Non-wavelet matrices can be used if the clustering or classification of the nucleotide sequences is of interest, without being precise about exact degree of scaling.

**2. In**Results and discussion*The advantage of the proposed method is that the scaling measure is invariant to the assignment of nucleotides. This implies that the method can capture any characteristics of nucleotides (e.g., amino and keto, purine and pyrimidine, and GC-content). Nevertheless, the authors showed only an example of intron-exon sequences in honeybee. As mentioned by the authors in*Background, *the exon sequences are correlated with GC-content. Therefore, it is validated that the method can capture the characteristics of the GC-content, but it can not be validated for the other characteristics. To claim the universality of the method, the authors should show that the method can be applied to any characteristics of nucleotides (e.g. purine and pyrimidine) by using the other example of biological sequences or simulated sequences.*

The scaling measures (slopes) indeed depend on the GC-content of nucleotides. However, we demonstrated through our permutation test that when GC-content is fixed, spectral slopes still differ for different sequences. In this paper, we have taken exons and introns as examples of types of sequences to be analyzed using our methodology. We plan to investigate other examples of sequences in the future.

**Minor comments 1.***In Fig.*8, *show the names of x-y axes.*

Thanks. Since these are estimators of the density, we opted not to burden image/description with new notation. Instead, the figure caption now reads: “Kernel density estimates for cumulative evolutionary slopes, shown on the horizontal axis, of exons (red) and introns (green).”

## Reviewer reports: round 2

### Response to referee Dr. Vladislav Bondarenko

Major comment: *First of all, the*Background*section improved notably after the first review, and we have no more questions to it. However, there are still open questions to the study design and methodology.However, I concern regarding a very low sensitivity (50%) and overall accuracy (64%) of the method. It is very limited hope that the method may be competitive with dozen alternative methods using in the field. Unfortunately, the authors still ignore several general (and basic) requirements to the methodological works, including the reproducibility analysis and the comparison with known methods. Many questions (including statistical tests and significance values) are still open. It is not easy what a benefit of the method for biological applications is.*

The authors appreciate time you invested in detailed reading and useful response. Below are our answers to your specific concerns 1-4.

*1. Methods. Equivalent classes. It remains unclear to me why authors use a paired nucleotide notation to introduce equivalent classes. Please explain. Is there any difference if we use a three (for example, genetic code) or four nucleotide notation?*

Equivalence classes are determined by the invariance of the slopes with respect to permutations of nucleotides. In this sense, the classes can be thought as mathematical objects and they are not predesigned. It happens that a particular class can be fully described by permutations of the pairs of nucleotides. Using groups of three nucleotides, for example, would not keep the slopes unchanged. Although this pairing is purely mathematical, there are some biological consequences. From the biological perspective, an equivalence class that is based on paired nucleotide notation is more suitable over a three or four nucleotide notation mainly because interesting classification schemes of nucleotide symbols (A, C, G, and T) based on nucleotide characteristics occur in pairs (purine-pyrimidine nucleotides, nucleotides with weak-strong hydrogen bonds, keto-amino nucleotides). Therefore, in addition to its purely mathematical generative process, the proposed method allows such physicochemical characteristics of nucleotides to be captured simultaneously. In addition, the use of paired nucleotide notation in equivalence class allows the interference of commonly found CG dinucleotides to be eliminated. (Previous works on biological sequence analysis often examine the correlation of GC content and its roles in genome/gene sequence variation, e.g., in genome/chromosome size, sequence functioning, gene architecture, species ecology, species evolution, etc.)

*2. Methods. Data. The authors don’t need a “completely annotated” genome it order to analyze 376 genes on a single chromosome. It is nonsense. Fruit fly or human genomes are annotated much better, providing evidences from RNA-Seq data and other sources and a proportion of protein-coding genes with CDS data support will be much higher than 376/1,669 (honeybee 1st chromosome).*

Probably there is a misunderstanding in the terminology use. In trying to assess the classification accuracy of the method, the annotation would mean that we know the ground truth. Of course, the method, once established on the training sample, would be applicable to data where the ground truth is not available. We selected honeybee genome to be our illustration dataset, partly because it has a complete genome and is considered a model organism. Genomes of other eukaryotic species could be selected as well, of course, but we simply did not analyze them.

*3. Methods. Data. “.. Specifically, we correct the gene direction and represent the gene sequences with their complementary sequences.” This step confused me a lot. Does it mean that authors take a complementary (non-coding) strand of a gene if it is located on the opposite strand? If so, it is most probably to be incorrect.*

Given the complementary directions of DNA string on its double helix structure, it is sufficient to represent a DNA molecule by the nucleotide sequence of a single strand. Sequence databases always store DNA sequences in the 5’ to 3’ direction and nucleotides are indexed in such fashion (1…*n*), regardless of the gene directions. In summary, when genes are located on the reverse strand, we reverse its complement sequence to keep the 5’ and 3’ ends properly oriented.

*4. Results. Figures *5*and*6. *I suggest it makes sense to separate intron/exon and exon/intron boundaries in the “combined set” of sequences, since most probably they would have a different (reverse) patterns of regularity.*

### Replies to the summary previous questions of reviewer 1

*Q1. What is an “overlapping section of nucleotides” remains not mentioned in the answer.*

The term “overlapping section of nucleotides” represents the spanning region of intron and exon. Our method used a window size of 32 nucleotides to calculate a cumulative slope. Therefore, the window that contains nucleotides of both exon and intron regions was defined as the “combination region”. Without defining the combination regions, the slopes were getting averaged for portions of exons and introns together which affected the results. Our rationale of introducing the “overlapping sections” was explained in the Results and discussion section, under the subsection “Comparing regularity of honeybee and simulated DNA.”

*Q9. Similarly. The significance is already provided in the main text, if I am correct.*

The significant difference in cumulative evolutionary slopes of simulated and genuine DNA arrays was mentioned in the main text, in the Results and discussion section, under the subsection “Comparing regularity of honeybee and simulated DNA.” The section states that both the global and the cumulative evolutionary slopes are influenced by the internal autocorrelations of nucleotides (sequence regularities), and unlikely by the proportional content of each nucleotide.

*Q11. The question is related to a global slope calculation, but it looks like the authors did not understand the question.*

The global slope measures the sequence regularity in a gene and ignores the sequence annotation (introns, exons). We used this slope to compare the overall regularity of a given honeybee gene vs. a set of simulated sequences (20,000 permutations). As the method of global calculation requires the sequence length to be a power of 2, each sequence was truncated accordingly. We empirically chose to truncate at the gene’s end. The average percentage of sequence length retained for global slope analysis across all 376 genes is 71.6 % (95 % confidence interval of 70.1–73.1 %). Truncation at the beginning of a gene is also possible but we did not do so. The reviewer commented that sequence truncation is also possible at other locations within a gene (at the beginning of a CDS, its end, or repeated loci). We did not examine such scenarios since (i) it is impractical to apply such setting to every gene in our gene set, and (ii) there is no way to ensure how many nucleotides to be removed per a sub-location to ensure the final gene length equals to 2^*n*^.

*Q13. At least two reviewers asked this question and it remains unclear to me why having 6 or 8 months already they haven’t done it on the other species (at least fly) yet, since one of their arguments was a “time constraint.”*

The aim of this paper was not to show the universal applicability of the proposed methodology to a range of species with decoded genome. We reiterate that its main contribution is *determination of scaling exponents in nucleotide sequences that are invariant with respect to assignment of particular nucleotides to numbers* and the honeybee genome was used as an illustration in the context of well understood phenomenon of different scaling in exons and introns. Thus the honeybee chromosome 1 was a showcase for the methodology.

*Q14. No. Looking at the Fig.*3, *I cannot conclude that the p-value is “extremely” small. It should be provided; otherwise this figure does not make sense.*

As we described in the manuscript, Fig. 3 shows the distribution of slopes for DNA-like sequences, randomly generated from the multinomial distribution where the probabilities of A, C, G, and T match those of the real exemplary strain of nucleotides from honeybee. The message is that irregularity of the random sequence exceeds that of the real DNA. There is no *p*-value here, for there is no test. Simply the histogram is a parametric bootstrap distribution of the slopes and the red bar is unusual for this distribution. What one could refer to as a “*p*-value” would be the Achieved Significance Level (ASL), the counterpart of *p*-value in bootstrap computations, and for this case the ASL is the proportion of the histogram to the left of the red bar, that is 0.0676.

To clarify our point, the sentence in explanation of Fig. 3:

“Comparing the actual and simulated DNA sequences, the actual DNA sequence’s slope falls in the left tail of the control distribution.”

is replaced by

“Comparing the actual and simulated DNA sequences, the actual DNA sequence’s slope falls in the left tail of the control distribution. The achieved significance level (ASL) for this simulation (area in the bootstrap distribution left of the red bar) is 0.0676”.

*Q15. It would be a good strategy to compare sensitivity/specificity with the analogous methods. But they won’t do it - most probably, it fails.*

The reviewer is probably right. Looking only on spectral/regularity information is likely suboptimal compared to a battery of state-of-art machine learning techniques for the corresponding classification. But this is not the ultimate point here. The goal of the paper was not to propose the most accurate classification method, but to emphasize phenomenology of different scalings in introns and exons captured by the proposed methodology. Even highly cited and famous paper of Peng et al. (1992) in *Nature*, that first established different scaling in exons and introns would be inadequate if the accuracy of classification was questioned, since its discriminatory power is low.

*Q16. This is a very weak and not direct criteria.*

If we understand correctly, this reviewer is pounding on the fact that the classifying accuracy in the illustrative example is rather weak. We do agree. But given a battery of strong classifiers, adding an independent and weak classifier to this battery improves overall classification accuracy. As the machine learning community jokingly states, *adding a new and independent classifier with accuracy better than flipping a coin, makes a strong classifier even stronger.*

### Response to referee Dr Natsuhiro Ichinose

*For Comment 2, My point is what the proposed method can do but the other methods cannot do. Since the DNA-walk method can detect irregularity of exons, this result cannot be the advantage even if the proposed method can detect it. In my understanding, the advantage is that the proposed method is the universal detector of sequence characteristics because the scaling measure is invariant. Therefore, I think that it is difficult to show the advantage of the proposed method by only a single example. At least, the authors should show the advantageous point against the conventional DNA-walk method (smaller window size, etc).*

Thank you for your time and constructive comment. You are right – the advantage of the proposed method is the invariance of the discriminatory spectral slope to the assignment of nucleotides to numbers which generates a random walk. We are not claiming that the proposed method is more accurate than a method that specifies the nucleotides, say purine and pyrimidine, for the increments of +1 or −1. If the purine/pyrimidine phenomenology is of concern – then, of course, one should use this specific methodology. However, it may be unsettling that different assignments produce different Hurst exponents/slopes for the same sequence, and the main goal of this paper is to remove this ambiguity.

Since the selection of honeybee genome is for the illustration for the methodology, we did not explore performance on other eukaryotic species. We do agree with the reviewer that this is an interesting question and a new paper can be devoted to a comparative assessment of the methodology. In such new comparative paper it would be of interest to compare invariant assignment with specific assignments such as purine-pyrimidine nucleotides, nucleotides with weak-strong hydrogen bonds, and keto-amino nucleotides for variety of species.

## Additional files

Additional file 1
**MATLAB function for producing the cumulative evolutionary slope for a DNA sequence.** (M 1.44 kb)

Additional file 2
**MATLAB function for producing plots of the cumulative evolutionary slope with plotted averages for coding, noncoding, and combination regions.** (M 3.94 kb)

Additional file 3
**Plotted results of the permutation test.** The histogram shows the permutation null distribution. The red bar is the slope for the un-permuted DNA string (empirical *p*-value =0.0638). (ESP 1.7 kb)

## Abbreviations

CDS: Coding sequence
DNA: Deoxyribonucleic acid
MCC: Matthews correlation coefficient
RNA: Ribonucleic acid
